# A Multicenter Randomized Phase II Study of Single Agent Efficacy and Optimal Combination Sequence of Everolimus and Pasireotide LAR in Advanced Thyroid Cancer

**DOI:** 10.3390/cancers14112639

**Published:** 2022-05-26

**Authors:** Julie E. Bauman, Zhengjia Chen, Chao Zhang, James P. Ohr, Robert L. Ferris, Gerald M. McGorisk, Stephen Brandt, Sumathi Srivatsa, Amy Y. Chen, Conor E. Steuer, Dong M. Shin, Nabil F. Saba, Fadlo R. Khuri, Taofeek K. Owonikoko

**Affiliations:** 1 Department of Medicine, Division of Hematology/Oncology, UPMC Hillman Cancer Institute, University of Pittsburgh, Pittsburgh, PA 15213, USA; jebauman@gwu.edu (J.E.B.); ohrj@upmc.edu (J.P.O.); 2 Winship Cancer Institute of Emory University, Atlanta, GA 30322, USA; znchen@uic.edu (Z.C.); chao.zhang2@emory.edu (C.Z.); dmshin@emory.edu (D.M.S.); nfsaba@emory.edu (N.F.S.); frkhuri@aub.edu.lb (F.R.K.); 3 Department of Biostatistics, Rollins School of Public Health and Winship Cancer Institute of Emory University, Atlanta, GA 30322, USA; 4 Department of Otolaryngology, Hillman Cancer Institute, University of Pittsburgh, Pittsburgh, PA 15213, USA; ferrrl@upmc.edu; 5 Department of Medicine, Division of Cardiology, Emory University, Atlanta, GA 30322, USA; gmcgori@emory.edu; 6 Department of Medicine, Division of Endocrinology, Emory University, Atlanta, GA 30322, USA; stephen.brandt@emoryhealthcare.org (S.B.); ssrivat@emory.edu (S.S.); 7 Department of Otolaryngology, Emory University, Atlanta, GA 30322, USA; achen@emory.edu; 8 Department of Hematology/Medical Oncology, Emory University, Atlanta, GA 30322, USA; csteuer@emory.edu

**Keywords:** thyroid cancer, somatostatin receptor, everolimus, pasireotide, combination, medullary carcinoma, TOR Serine–Threonine Kinases

## Abstract

**Simple Summary:**

Prior clinical studies showed modest activity for single agent everolimus and somatostatin analogues in different subtypes of thyroid cancer; the combination of everolimus and somatostatin analogue was synergistic in preclinical models of thyroid cancer. This randomized trial showed that the combination was more effective than a single agent and the sequence of single agent everolimus followed by the delayed combination of everolimus and pasireotide-LAR achieved the best efficacy and was the optimal combination strategy.

**Abstract:**

Purpose: Aberrant mTOR pathway and somatostatin receptor signaling are implicated in thyroid cancer and offer potential therapeutic targets. We assessed the clinical efficacy of everolimus and Pasireotide long-acting release (LAR) in radioiodine-refractory differentiated thyroid cancer (DTC) and medullary thyroid cancer (MTC). Patients and methods: Adults with progressive MTC and DTC untreated or treated with no more than one systemic agent were eligible. The trial was designed to establish the most promising regimen and the optimal combination sequence. Patients were randomized to start treatment with single agent everolimus (10 mg QD; Arm A), pasireotide-LAR (60 mg intramuscular injection, Q4 weeks; Arm B), or the combination (Arm C). At initial progression (PFS1), patients on Arm A or B switched to the combination and continued until progression (PFS2). Efficacy was measured by RECIST criteria. Results: Study enrolled 42 patients: median age 65 years; female 17 (40.5%); White 31 (73.8%), African American 6 (14.3%), others 5 (11.9); DTC 32 (76.2%); MTC 10 (23.8%). There was no objective response by RECIST criteria across the three arms. Median and 1-year PFS1 rates were 8.3, 1.8, 8.1 months and 49.9%, 36.4%, 25.0% for Arms A, B, C, respectively. Median and 1-year PFS2 rates were 26.3, 17.5, 8.1 months and 78.4%, 70.0%, 25% for Arms A, B, C, respectively. The most frequent adverse events were anemia, stomatitis, fatigue, hyperglycemia, and hypercholesterolemia. Conclusions: The combination of everolimus and pasireotide-LAR showed promising efficacy over single agent. The delayed combination of everolimus and pasireotide-LAR following progression on single agent everolimus appeared intriguing as a combination strategy.

## 1. Introduction

Advanced thyroid cancer remains a significant challenge with limited therapeutic options. Lenvatinib, cabozantinib, vandetanib, and sorafenib have established efficacy, leading to regulatory approval [1,2,3,4]. However, the disease control achievable with these agents lasts less than 2 years on average. Despite the implicated role for somatostatin receptor activity in thyroid proliferation, anecdotal reports of treatment with somatostatin analogues, octreotide, and lanreotide in differentiated thyroid cancer patients showed quite modest efficacy [5,6,7,8]. Pasireotide is a new generation somatostatin analogue that targets four of the five known isoforms of the somatostatin receptor [9,10,11]. This broad activity is expected to result in a greater antitumor effect compared to subtype 2 selective somatostatin analogues like lanreotide and octreotide.

Isolated inhibition of the mTOR pathway through downstream blockade with rapamycin analogues engenders a compensatory activation of survival pathway response with increased signaling through AKT, MEK/ERK, and EGFR [12,13,14,15]. Conversely, a dual blockade of the mTOR and alternative survival pathways results in increased efficacy [12,13,14]. The combination of somatostatin analogue octreotide and mTOR inhibitor everolimus led to greater efficacy in preclinical models of thyroid cancer and clinically in pancreatic neuroendocrine tumors [16,17,18]. Because somatostatin analogue sensitizes tumor cells to mTOR inhibition and can also reverse resistance to mTOR inhibition through its inhibition of Akt [19,20], we expect that the combination of pasireotide long-acting release (LAR) and everolimus will result in even greater antitumor efficacy. It remains unclear, however, whether initial mTOR inhibitor therapy to first induce cellular addiction to the compensatory survival pathway is superior to the reverse schedule of blocking the survival pathway prior to mTOR inhibition. Additionally, the superiority of either of these schedules over simultaneous inhibition with combined mTOR and, for instance, a PI3/Akt inhibitor is yet to be established in the clinical setting.

This trial was designed to demonstrate clinical single agent activity of everolimus and pasireotide LAR. It also sought to establish whether efficacy is best improved by the immediate combination of both agents versus delayed combination where patients starting with a single agent received the combination of both agents at the time of disease progression.

## 2. Materials and Methods

The primary objective of this phase II trial was to compare the efficacy of everolimus, pasireotide LAR, and their combination in radioactive iodine (RAI)-refractory differentiated thyroid cancer (DTC) and medullary thyroid cancer (MTC) using two co-primary endpoints, objective response rate (ORR), and progression-free survival (PFS). The study was approved by the respective Institutional Review Board (IRB) at both participating sites. Study participants provided a written informed consent prior to undergoing any study-related procedures. The study was registered at www.clinicaltrials.gov with the reference NCT01270321.

Patients were eligible for this trial if they were ≥18 years of age with histologic diagnosis of thyroid cancer (papillary, follicular, and medullary) that had progressed within the previous 12 months. Patients with DTC were required to have refractory disease or not be eligible for further treatment with RAI as defined by one of the following criteria: no iodine uptake on a post-RAI treatment scan (obtained in the presence of low iodine diet and adequate thyroid stimulating hormone (TSH) stimulation) in an anatomically defined lesion that qualified as target lesion by the Response Evaluation Criteria in Solid Tumours (RECIST) criteria (if there was demonstrable iodine uptake, the last RAI treatment (≥100 mCi) was given within the prior 16 months or if given more than 16 months before enrollment, there was evidence of disease progression after each of the previous two RAI treatments performed within 16 months of each other); maximum cumulative lifetime dose of RAI treatments of ≥600 mCi; intolerant of RAI or with heavy disease burden that in the opinion of the treating physician is not likely to benefit from further RAI. Other eligibility criteria were the presence of at least one site of measurable disease, according to RECIST criteria version 1.1; good performance status of 0–2 on the Eastern Cooperative Oncology Group (ECOG) scale; adequate organ function as indicated by absolute neutrophil count (ANC) ≥1.5 × 10^9^/L, platelets count ≥100 × 10^9^/L, hemoglobin >9 g/dL; serum bilirubin ≤1.5 × upper limits of normal (ULN), serum transaminases ≤3 × ULN, and serum creatinine ≤1.5 × ULN.

Reasons for exclusion from the study included prior treatment with systemic agent (this was later amended to allow for no more than 1 prior systemic treatment); a condition that led to impairment of gastrointestinal function that may have altered the absorption of everolimus; chronic treatment with systemic steroids or other immunosuppressive agents; uncontrolled diabetes mellitus or elevated fasting plasma glucose >1.5 ULN; symptomatic cholelithiasis or risks of Torsades de Pointes, including prolonged QTcF >470 millisecond, clinically significant cardiac arrhythmias, and a history of syncope or family history of idiopathic sudden death.

**Figure 1:**
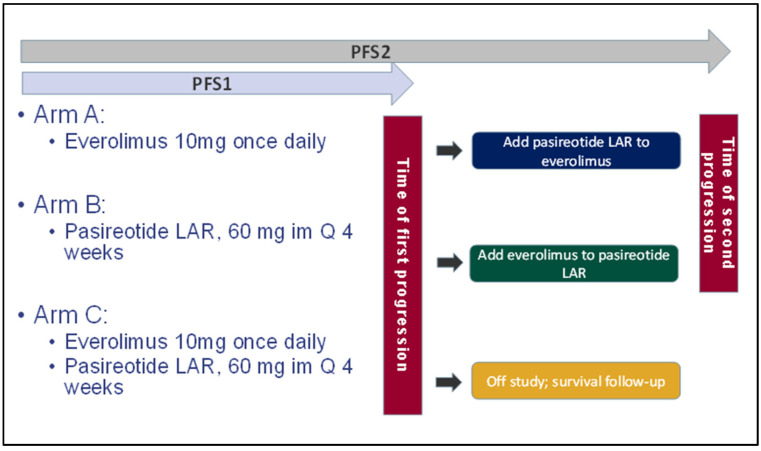
Randomization of patients enrolled on study to single agent everolimus, pasireotide LAR, or the combination. PFS1 was measured from initiation of treatment until the time of initial progression. PFS2 is the interval from treatment initiation until progression on the combination regimen. Note that PFS1 is the same as PFS2 for Arm C.

*Study design*: The study was designed as an open-label, 3-arm, randomized phase II clinical trial to study the single agent and combined efficacy of pasireotide LAR and everolimus in thyroid cancer patients (Figure 1). Patient randomization was stratified by histology (medullary versus non-medullary thyroid cancer). To explore the optimal schedule of administration, patients who were initially randomized to a single agent arm were allowed to receive the second agent in combination with the initial agent at the time of disease progression.

*Treatment*: Everolimus was administered at a standard dose of 10 mg once daily, orally, continuously on days 1 to 28 of a scheduled 28-day cycle. Pasireotide LAR was given as an intramuscular injection 60 mg once on day 1 of each cycle. Patients remained on treatment until evidence of disease progression, intolerable toxicity, or withdrawal of consent.

*Statistical considerations*: The study design was a randomized screening design with three arms, and the arm that met the prespecified efficacy threshold would be picked as the winner for further clinical testing in a future trial. Randomization was conducted with blocks of 3 patients to balance the enrollment in each arm. Within each of the 3 arms, a Simon’s 2-stage MinMax design was used. The null hypothesis was that the response rate for an ineffective treatment regimen would be less than 5%. The alternative hypothesis was a response rate >15% if the treatment was effective. The study power was 80% and the significance level was at 20%. In each arm, 18 evaluable patients would be enrolled into stage I and if there was at least one objective response, the study would proceed to the stage II accrual of 10 additional patients for a total of 28 patients per arm. At least three out of 28 patients were required to achieve objective response in order for the specific treatment regimen to be considered worthy of further clinical evaluation. In the event of identical ORR between the arms or if none of the arms met the ORR criterion (less than 3 objective responses after full patient enrolment), the 1-yr PFS rate would be employed to determine a winner.

Based on trials of targeted agents in thyroid cancer patient populations showing median PFS ranging 9–18 months [1,2,3,4], only 20% of patients were expected to be progression-free at 1 year if the treatment was ineffective, while ≥50% of the patients would be progression-free at the same time point with an effective regimen. For this purpose, a 1-year PFS rate of ≥50% would be considered sufficient activity to justify further evaluation of the treatment in this disease. With this assumption, 28 patients enrolled onto each arm would be needed to classify the treatment as having sufficient activity for further evaluation (50% under alternative hypothesis vs. 20% under null hypothesis) with a power of 0.95 at the significance level of 0.05.

Descriptive statistics for each variable were reported. For numeric covariates, the mean and standard deviations were calculated and presented. Frequency and percentage were shown for categorical variables. Fisher’s exact test or Chi-square test was used to detect differences for categorical covariates, and ANOVA test was used for numerical covariates. PFS1 was calculated as time from randomization to initial disease progression on the initial treatment assigned at randomization. PFS2 was calculated as the time from randomization to the second progression for patients on Arm A or B after switching to the combination of the two investigational agents. The univariate association of each covariate with OS or PFS was assessed using the Cox proportional hazards model. KM plot and log-rank test were presented for OS, PFS for some covariates. Statistical analysis was conducted using SAS Version 9.4 and SAS macros developed by the Biostatistics and Bioinformatics Shared Resource at Winship Cancer Institute.

## 3. Results

### 3.1. Patient and Tumor Characteristics

The study enrolled a total of 42 patients, including 17 females (40.5%) and 25 males (59.5%) with advanced stage thyroid cancer who were eligible for the initiation of a targeted biologic agent because of disease progression. At the time of manuscript writing, 10 patients were alive and none remained on active treatment. Details of patient demographics and disease characteristics at the time of registration by treatment arm are summarized in Table 1. Patient distribution across the three arms was comparable by ethnicity, histology, and age but not by race; African American patients were randomized only to single agent Arms A and B but not to the combination Arm C.

### 3.2. Efficacy

Due to the challenges of subject accrual as new treatment options for thyroid cancer became available during the course of this study, a protocol amendment was effected that allowed us to conduct an early interim analysis for efficacy prior to enrolling 18 patients in stage I as initially planned. The interim analysis was conducted based on Simon’s two stage MinMax design to test for promising efficacy signals based on at least one patient with objective response and in the absence of a responder to test if the 1-year PFS rate was >50%. At the time of this analysis, there were 11 patients on Arm A, 11 patients on Arm B, and 12 patients on Arm C. No patient met RECIST-defined objective response on any of the study arms at the time of the interim analysis. Therefore, the PFS endpoint was employed to establish whether any of the three arms was worthwhile to study further. The 1-year PFS1 rate counting from treatment initiation to initial progression on assigned treatment was 54.5% (22.9%, 78.0%), 43.6% (14.7%, 69.9%), and 50.0% (18.4%, 75.3%) for arms A, B, and C, respectively. Similarly, the 1-year PFS2 rate, counting from treatment initiation until progression on the combination treatment for those starting with single agent, was 90.9% (50.8%, 98.7%), 61.4% (26.6%, 83.5%), and 50.0% (18.4%, 75.3%), respectively, for Arms A, B, and C. Arm A was therefore deemed the most promising arm of the study leading to enrollment discontinuation to arms B and C after the interim analysis.

**Figure 2:**
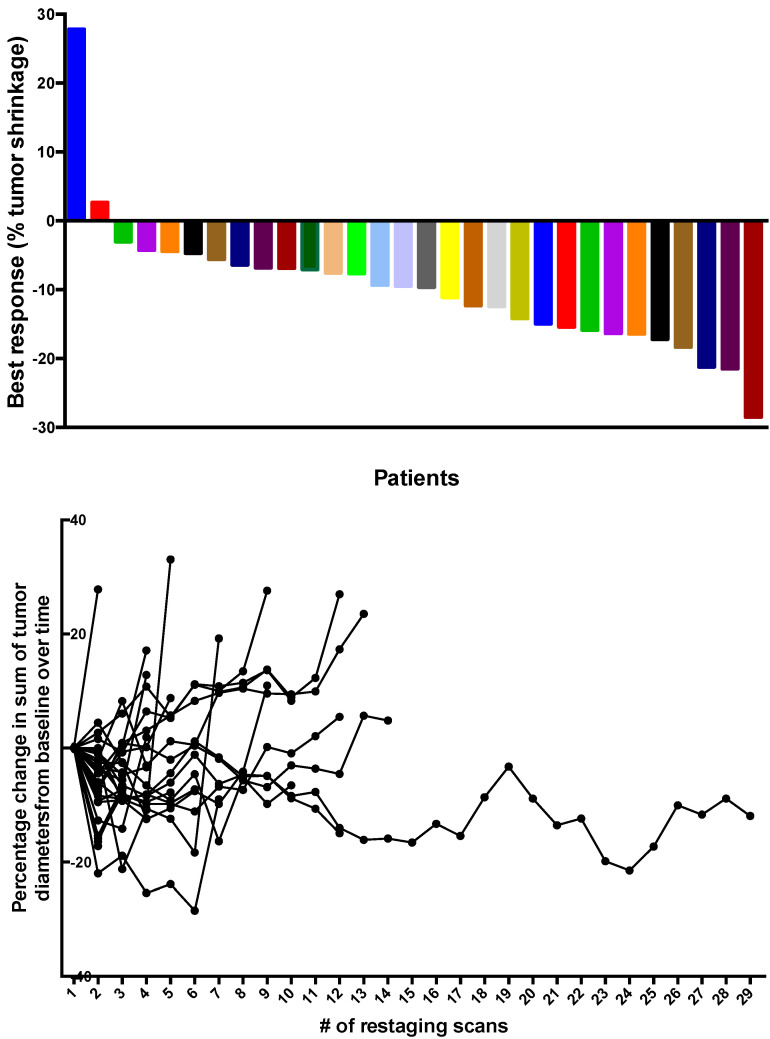
Top: Waterfall plot showing the best response on study measured as percentage change in sum of tumor diameters compared to baseline. Bottom: Spider plot of changes in tumor burden over time measured as percent change in the sum of target lesion diameters compared to baseline measurement at serial timepoints, corresponding to restaging scans obtained after every 2 cycles (8 weeks) for patients on the study. There was no patient meeting the objective response criteria according to RECIST 1.1 criteria (i.e., no patient experienced a tumor shrinkage of 30% or greater in the sum of the tumor target lesion diameters compared to baseline measurements) and the majority of patients achieved stable disease as best outcome.

**Figure 3:**
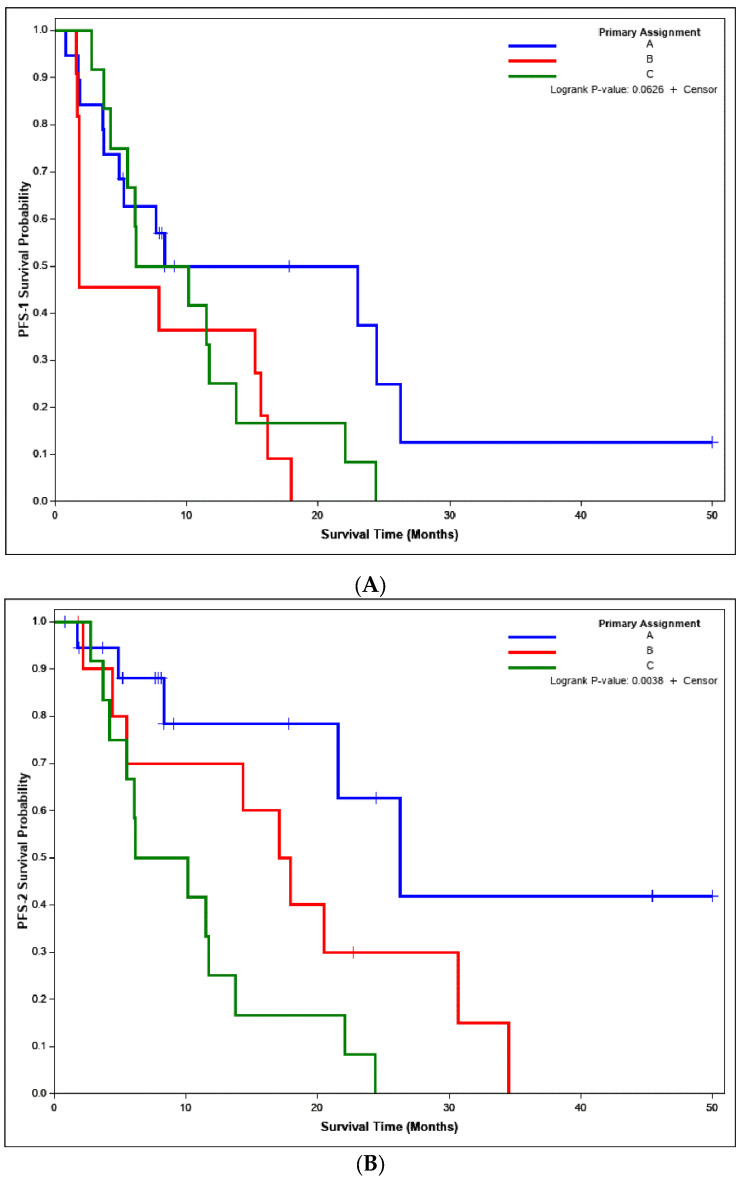
Comparison of PFS between the three arms of the study. (**A**). PFS1 from treatment initiation until initial progression of disease or death; (**B**). PFS2 calculated across initial and second disease progression while on combination treatment or death. Comparable median PFS1 for Arms A and C at 8.3 and 8.1 months but only 1.8 months for Arm B. The 1-year PFS1 rate was 49.9% (25.2%, 70.4%) for arm A but below the prespecified efficacy threshold of 50% for arm B at 36.4% (11.2%, 62.7%), and arm C at 25.0% (6.0%, 50.5%). PFS2 was significantly prolonged in arms A and B in comparison to arm C. Median PFS2 was 26.3 (8.3, NA) months for arm A and 17.5 (2.1, 30.7) months for arm B but only 8.1 (3.7, 13.8) months for patients on arm C. The 1-year PFS2 rate was also higher at 78.4% (44.9%, 92.8%) and 70.0% (32.9%, 89.2%) for arm A and B, respectively, compared to 25.0% (6.0%, 50.5%) for Arm C.

At the final efficacy analysis with 42 enrolled patients (19 patients on Arm A treated with everolimus, 11 patients on Arm B treated with pasireotide LAR, and 12 patients on Arm C treated with the combination of both agents) no patient achieved an objective response by RECIST criteria but almost all the patients experienced varying degrees of tumor shrinkage (Figure 2). The 1-year PFS1 rate with the mature data was 49.9% (95% CI: 25.2%, 70.4%) for single agent everolimus, 36.4% (95% CI: 11.2%, 62.7%) for pasireotide LAR, and 25.0% (6.0%, 50.5%) for the combination. The median PFS1 was not significantly different between the arms at 8.3 months (95% CI: 3.7, 26.3), 1.8 months (95% CI: 1.7, 15.6), and 8.1 months (95% CI: 3.7, 13.8) (*p* = 0.0626) for everolimus, pasireotide LAR, and the combination, respectively (Figure 3). Conversely, the 1-year PFS2 rate was significantly higher for patients in Arms A and B at 78.4% (95% CI: 44.9%, 92.8%) and 70.0% (95% CI: 32.9%, 89.2%) compared to 25.0% (95% CI: 6.0%, 50.5%) for Arm C. Median PFS2 was significantly different between the arms at 26.3 months (95% CI: 8.3, NA), 17.5 months (95% CI: 2.1, 30.7), and 8.1 months (95% CI: 3.7, 13.8) (*p* = 0.0038) for Arms A, B, and C, respectively (Figure 3). While the median overall survival (OS) was numerically longer for patients enrolled on Arms A and B, it was not significantly different between the three arms of the study at 41.6 months (95% CI: 17.8, NA) for Arm A, 39.4 months (95% CI: 8.4, NA) for Arm B, and 24.3 months (95% CI: 13.1, NA) for Arm C (*p*= 0.3067) (Figure S1).

Subset analysis was performed to assess whether the significant difference in PFS2 was driven by any patient subgroup. There were no significant differences in median PFS1 and PFS2 between patient subgroups defined by race (Supplementary Figure S2), gender (Supplementary Figure S3), or histologic subtype of thyroid cancer (Supplementary Figure S4). Median PFS2 comparison by race: 20.5 months (95% CI: 5.5, NA) for African Americans versus 11.7 (95% CI: 6.2, 21.6 for Whites (*p* = 0.3242); by gender: 11.5 months (95% CI: 4.8, 18) for females versus 21.6 months (95% CI: 8.3, 26.3) for males (*p* = 0.1797); and by histology: 18 months (95% CI: 10.1, 26.3) for DTC compared to 13.8 months (95% CI: 3.7, 21.6) for MTC (*p* = 0.1051).

### 3.3. Adverse Events

The most frequently reported treatment-emergent adverse events graded according to Common Terminology Criteria for Adverse Events (CTCAE) version 4.0 on study were hypercholesterolemia, anemia, and thrombocytopenia. Hyperglycemia was the most frequently encountered grade ≥3 adverse event. Full details of treatment-related and grade ≥3 adverse events are displayed in Table 2.

## 4. Discussion

Targeted biologic agents are now established therapeutic modalities for patients with MTC and RAI-refractory DTC [1,2,3,4]. Nonetheless, additional options are needed to improve on the current standard agents. The four approved kinase inhibitors for the treatment of MTC and DTC share a common feature of potent antiangiogenic activity [1,2,3,4]. Vascular toxicity associated with these agents poses a particular clinical challenge and limits the benefits of therapy only to patients without significant cardiovascular comorbidities, who are able to withstand the on-target toxicity of these agents. Other aberrant signaling pathways are implicated in the development of thyroid cancer [21,22,23]. For instance, aberrant signaling in the PI3K/AKT/mTOR pathways is directly implicated as contributing to the development of MTC and DTC [22,24,25,26]. Additionally, based on the high expression of somatostatin receptor in thyroid cancer, somatostatin analogues are expected to have clinical benefit in thyroid cancer [18]. Indeed, octreotide and lanreotide, which have strong affinity for somatostatin receptor subtype 2, were previously explored for the treatment of advanced thyroid cancer and showed modest clinical activity in the form of biochemical response and symptomatic relief [5,6,7,8,27,28]. The broad activity of pasireotide against the somatostatin receptor subtypes 1, 2, 3, and 5 was expected to translate into better clinical efficacy against neuroendocrine tumors. In addition to its known direct effect on somatostatin signaling, pasireotide can also inhibit Akt signaling, which should counteract the paradoxical Akt elevation that mediates resistance to mTOR inhibitors in preclinical experiments. We previously showed in preclinical models of thyroid cancer that the combination of pasireotide LAR and everolimus was superior to each agent alone [18]. This clinical study explored the clinical efficacy of two biologic agents, which are not direct antiangiogenic agents, with the expectation that a promising signal of efficacy would lead to the development of alternative treatment options for thyroid cancer patients who may not be candidates for antiangiogenic agents.

Similar to other smaller studies of everolimus, we observed only modest clinical activity for single agent everolimus. The median PFS was 8.3 months, which is comparable to the median PFS of 8–12 months previously reported in DTC and MTC patients [29,30,31]. While there is no previous report of clinical efficacy of single agent pasireotide LAR in thyroid cancer patients, the clinical activity observed in this study with a median PFS of 1.8 months and 1-year PFS rate of 36% is negligible and not significantly better than the clinical activity observed with lanreotide or octreotide in thyroid cancer patients [6,7,8].

The concurrent combination of everolimus and the long-acting somatostatin analogue, sandostatin, is an effective treatment in pancreatic neuroendocrine tumors. However, the optimal sequencing of this combination strategy has not been previously tested in patients. In the current study, the immediate combination of everolimus and pasireotide LAR did not appear to be significantly better than single agent everolimus, with a median PFS of 8.1 and 8.3 months, respectively. Indeed, the 1-year PFS rate was numerically higher at 49.9% for single agent everolimus versus 25.0% for the combination, in part because of early discontinuation of the combination therapy due to treatment-related toxicity. Interestingly, the sequential combination strategy where patients who started everolimus as a single agent and later switched to the combination of both drugs at the time of progression appeared to be a more effective strategy based on the PFS2 rate, which measured overall efficacy from initial treatment until disease progression in the subset of patients who received combination treatment after initially progressing on single agent (median PFS2 of 26.3 vs. 8.1 months and 1-year PFS2 rate of 78.4% vs. 25%). We observed a similar prolongation of overall clinical efficacy in patients who started on pasireotide LAR and later received the combination at progression, with a median PFS2 of 17.5 months and 1-year PFS2 rate of 70%. However, this sequence of pasireotide LAR followed by the combination was less effective than the sequence of single agent everolimus followed by the combination. Moreover, there was a rapid progression of disease on single agent pasireotide LAR with a median PFS of 1.8 months and a 1-year PFS rate of 39.4%, which did not meet the pre-specified threshold for a promising efficacy signal. These factors make the sequence of pasireotide LAR followed by combination a less appealing strategy for future development.

It is possible that sequential treatment with single agent everolimus and pasireotide LAR rather than their combination at the time of progression could have resulted in a similar outcome. However, the limited activity of pasireotide LAR in Arm B of the study argues against such an outcome. Indeed, the fact that the sequence of everolimus followed by the combination was the most promising is consistent with preclinical modeling studies in which paradoxical Akt activation limits anticancer efficacy of rapamycin and other allosteric mTOR inhibitors like everolimus. This Akt elevation could be reversed by the combination of a PI3K inhibitor and rapalogs [13]. Although pasireotide LAR is not a classical PI3K inhibitor, it has a potent inhibitory effect on activated Akt, which could therefore reverse the acquired resistance to everolimus, leading to the further prolongation of the clinical benefit of everolimus [20]. It is conceivable that pasireotide LAR has no impact on other potential resistance mechanisms at play in patients who started with the combination regimen, for whom the efficacy was no better than single agent everolimus. With the increasing number of targeted agents available for the treatment of thyroid and other types of cancer, treatment sequencing and the potential impact of combination strategies deserve greater attention. Our study showed a promising signal of improved efficacy with the sequential combination strategy over immediate combination of everolimus and pasireotide LAR and could inform the evaluation of similar strategies in other tumor types.

Some of the limitations of these findings include the fact that our study enrolled both MTC and DTC, whereas current therapeutic strategies in thyroid cancer approach MTC and DTC as different disease entities. Nonetheless, our findings are still informative and will be useful to drive the development of this strategy in both tumor types. Although the majority of enrolled patients had DTC, approximately one third had MTC. However, there was no significant difference in efficacy outcome based on tumor histology (median PFS of 10.1 vs. 6.7 months; *p* = 0.2199 and 1-year PFS rate of 41.3% vs. 26.7%, respectively, for DTC and MTC). Genomic profiling could have provided additional insights into the study result. However, we did not have such data because genomic profiling was not the standard of care for thyroid cancer as of the time this study was designed. We also could not reach the original accrual target proposed for the trial due to changing treatment landscape. The power consideration and corresponding targeted sample size calculation were based on a very high projected response rates which turned out to be overly ambitious. The trial was stopped prematurely before reaching the original target accrual. Notwithstanding the much smaller sample size, we still observed some interesting signals of efficacy. However, since the study is significantly underpowered, the results and any conclusions should be considered preliminary, and it is anticipated that future studies will provide further validation of this therapeutic strategy.

## 5. Conclusions

In conclusion, this signal-finding study showed some promise for the combination of everolimus and pasireotide-LAR, and a future confirmatory study is warranted. The strategy of the delayed evaluation of the 2-drug combination after the failure of single agent everolimus appeared intriguing and is worthy of further testing in the future in this disease and other cancer types.

## Figures and Tables

**Table 1:** Patient demographics, tumor characteristics, and distribution by treatment arm.

Covariate		Arm A N = 19	Arm B N = 11	Arm C N = 12
Race	White	15 (88.24)	5 (55.56)	11 (100)
	AA	2 (11.76)	4 (44.44)	0 (0)
Ethnicity	Hispanic	0 (0)	1 (10)	2 (18.18)
	Non-Hispanic	18 (100)	9 (90)	9 (81.82)
Histology	Differentiated	14 (73.68)	9 (81.82)	9 (75)
	Medullary	5 (26.32)	2 (18.18)	3 (25)
Age (mean ± SD)		66.95 ± 10.25	64.73 ± 12.55	58.83 ± 11.88

Arm A: Everolimus; Arm B: Pasireotide LAR; Arm C: Pasireotide LAR + Everolimus. At the time of the interim analysis there were 11, 11, and 12 patients in Arms A, B, and C, respectively. Eight additional patients were subsequently enrolled to Arm A, which was deemed the winning arm based on the 1-year PFS rate at interim analysis.

**Table 2:** Treatment emergent adverse events by treatment arm showing the most common toxicities and the highest-grade toxicities.

Arm A (N = 19)			
Most Frequent Adverse Events of Any Grade Occurring in >20% of Patients		Grade ≥3 Adverse Events in ≥5% of Patients	
Dry Skin	21%	Peripheral Edema	5%
Dysgeusia	21%	Fatigue	5%
Hypoalbuminemia	21%	Gastric Hemorrhage	11%
Joint Pain	21%	Anemia	5%
Cough	26%	Hypertension	5%
Diarrhea	26%	Hypocalcemia	5%
Hypercholesterolemia	26%	Leukopenia	5%
Hypertriglyceridemia	26%	Pneumonitis	11%
Hypokalemia	26%		
Hyponatremia	26%		
Mucositis	26%		
Rash	26%		
Thrombocytopenia	58%		
**Arm B (N = 11)**			
**Most Frequent Adverse Events of Any Grade Occurring in >20% of Patients**		**Most Frequent Grade ≥3 Adverse Events**	
Hyperbilirubinemia	27%	Blindness	9%
Hypertriglyceridemia	27%	Blood infection	9%
Hypercholesterolemia	36%	Dyspnea	9%
		Hypertension	9%
		Hyperglycemia	27%
**Arm C (N = 12)**			
**Most Frequent Adverse Events of Any Grade Occurring in >20% of Patients**		**Most Frequent Grade ≥3 Adverse Events**	
Hyponatremia	25%	Elevated GGT	8%
Mucositis	25%	Hyperglycemia	25%
Cough	33%	Hypokalemia	8%
Decreased Appetite	33%	Kidney stone	8%
Headache	42%	Mucositis	8%
Anemia	58%	Pain	8%
Hypercholesterolemia	75%	Hypertriglyceridemia	8%

All adverse events were graded according to CTCAE version 4.0.

## Data Availability

The data presented in this study are available on request from the corresponding author.

## Supplementary Materials

The following supporting information can be downloaded at: https://www.mdpi.com/article/10.3390/cancers14112639/s1, Figure S1: Overall survival by Arm of study; Figure S2: Progression-free survival by race/ethnic background; Figure S3: Progression-free survival by gender; Figure S4: Progression-free survival by tumor histology.

## References

[1] Brose M.S., Nutting C.M., Jarzab B., Elisei R., Siena S., Bastholt L., de la Fouchardiere C., Pacini F., Paschke R., Shong Y.K. (2014). Sorafenib in radioactive iodine-refractory, locally advanced or metastatic differentiated thyroid cancer: A randomised, double-blind, phase 3 trial. Lancet.

[2] Schlumberger M., Tahara M., Wirth L.J., Robinson B., Brose M.S., Elisei R., Dutcus C.E., de las Heras B., Zhu J., Habra M.A. (2014). A phase 3, multicenter, double-blind, placebo-controlled trial of lenvatinib (E7080) in patients with ^131^I-refractory differentiated thyroid cancer (SELECT). J. Clin. Oncol..

[3] Wells S.A., Robinson B.G., Gagel R.F., Dralle H., Fagin J.A., Santoro M., Baudin E., Elisei R., Jarzab B., Vasselli J.R. (2012). Vandetanib in patients with locally advanced or metastatic medullary thyroid cancer: A randomized, double-blind phase III trial. J. Clin. Oncol..

[4] Elisei R., Schlumberger M.J., Muller S.P., Schoffski P., Brose M.S., Shah M.H., Licitra L., Jarzab B., Medvedev V., Kreissl M.C. (2013). Cabozantinib in progressive medullary thyroid cancer. J. Clin. Oncol..

[5] Frank-Raue K., Ziegler R., Raue F. (1993). The use of octreotide in the treatment of medullary thyroid carcinoma. Horm. Metab. Res. Suppl..

[6] Kohlfuerst S., Igerc I., Gallowitsch H.J., Gomez I., Kresnik E., Matschnig S., Lind P. (2006). Is there a role for sandostatin treatment in patients with progressive thyroid cancer and iodine-negative but somatostatin-receptor-positive metastases?. Thyroid. Off. J. Am. Thyroid. Assoc..

[7] Mahler C., Verhelst J., de Longueville M., Harris A. (1990). Long-term treatment of metastatic medullary thyroid carcinoma with the somatostatin analogue octreotide. Clin. Endocrinol..

[8] Vainas I., Koussis C., Pazaitou-Panayiotou K., Drimonitis A., Chrisoulidou A., Iakovou I., Boudina M., Kaprara A., Maladaki A. (2004). Somatostatin receptor expression in vivo and response to somatostatin analog therapy with or without other antineoplastic treatments in advanced medullary thyroid carcinoma. J. Exp. Clin. Cancer Res. CR.

[9] Schmid H.A. (2008). Pasireotide (SOM230): Development, mechanism of action and potential applications. Mol. Cell. Endocrinol..

[10] Schmid H.A., Silva A.P. (2005). Short- and long-term effects of octreotide and SOM230 on GH, IGF-I, ACTH, corticosterone and ghrelin in rats. J. Endocrinol. Investig..

[11] Bruns C., Lewis I., Briner U., Meno-Tetang G., Weckbecker G. (2002). SOM230: A novel somatostatin peptidomimetic with broad somatotropin release inhibiting factor (SRIF) receptor binding and a unique antisecretory profile. Eur. J. Endocrinol. Eur. Fed. Endocr. Soc..

[12] Wang X., Hawk N., Yue P., Kauh J., Ramalingam S.S., Fu H., Khuri F.R., Sun S.Y. (2008). Overcoming mTOR inhibition-induced paradoxical activation of survival signaling pathways enhances mTOR inhibitors’ anticancer efficacy. Cancer Biol. Ther..

[13] Sun S.Y., Rosenberg L.M., Wang X., Zhou Z., Yue P., Fu H., Khuri F.R. (2005). Activation of Akt and eIF4E Survival Pathways by Rapamycin-Mediated Mammalian Target of Rapamycin Inhibition. Cancer Res..

[14] Wang X., Yue P., Kim Y.A., Fu H., Khuri F.R., Sun S.Y. (2008). Enhancing mammalian target of rapamycin (mTOR)-targeted cancer therapy by preventing mTOR/raptor inhibition-initiated, mTOR/rictor-independent Akt activation. Cancer Res..

[15] Moreno A., Akcakanat A., Munsell M.F., Soni A., Yao J.C., Meric-Bernstam F. (2008). Antitumor activity of rapamycin and octreotide as single agents or in combination in neuroendocrine tumors. Endocr.-Relat. Cancer.

[16] Cerovac V., Monteserin-Garcia J., Rubinfeld H., Buchfelder M., Losa M., Florio T., Paez-Pereda M., Stalla G.K., Theodoropoulou M. (2010). The somatostatin analogue octreotide confers sensitivity to rapamycin treatment on pituitary tumor cells. Cancer Res..

[17] Pavel M.E., Hainsworth J.D., Baudin E., Peeters M., Horsch D., Winkler R.E., Klimovsky J., Lebwohl D., Jehl V., Wolin E.M. (2011). Everolimus plus octreotide long-acting repeatable for the treatment of advanced neuroendocrine tumours associated with carcinoid syndrome (RADIANT-2): A randomised, placebo-controlled, phase 3 study. Lancet.

[18] Owonikoko T.K., Zhang G., Lallani S.B., Chen Z., Martinson D.E., Khuri F.R., Lonial S., Marcus A., Sun S.Y. (2019). Evaluation of preclinical efficacy of everolimus and pasireotide in thyroid cancer cell lines and xenograft models. PLoS ONE.

[19] Murasawa S., Kageyama K., Sugiyama A., Ishigame N., Niioka K., Suda T., Daimon M. (2014). Inhibitory effects of SOM230 on adrenocorticotropic hormone production and corticotroph tumor cell proliferation in vitro and in vivo. Mol. Cell. Endocrinol..

[20] Theodoropoulou M., Stalla G.K. (2013). Somatostatin receptors: From signaling to clinical practice. Front. Neuroendocrinol..

[21] Ernani V., Kumar M., Chen A.Y., Owonikoko T.K. (2016). Systemic treatment and management approaches for medullary thyroid cancer. Cancer Treat. Rev..

[22] Cancer Genome Atlas Research N. (2014). Integrated genomic characterization of papillary thyroid carcinoma. Cell.

[23] Prasad M.L., Vyas M., Horne M.J., Virk R.K., Morotti R., Liu Z., Tallini G., Nikiforova M.N., Christison-Lagay E.R., Udelsman R. (2016). NTRK fusion oncogenes in pediatric papillary thyroid carcinoma in northeast United States. Cancer.

[24] Matson D.R., Hardin H., Buehler D., Lloyd R.V. (2017). AKT activity is elevated in aggressive thyroid neoplasms where it promotes proliferation and invasion. Exp. Mol. Pathol..

[25] Moraitis D., Karanikou M., Liakou C., Dimas K., Tzimas G., Tseleni-Balafouta S., Patsouris E., Rassidakis G.Z., Kouvaraki M.A. (2014). SIN1, a critical component of the mTOR-Rictor complex, is overexpressed and associated with AKT activation in medullary and aggressive papillary thyroid carcinomas. Surgery.

[26] Tamburrino A., Molinolo A.A., Salerno P., Chernock R.D., Raffeld M., Xi L., Gutkind J.S., Moley J.F., Wells S.A., Santoro M. (2012). Activation of the mTOR pathway in primary medullary thyroid carcinoma and lymph node metastases. Clin. Cancer Res. Off. J. Am. Assoc. Cancer Res..

[27] Vitale G., Tagliaferri P., Caraglia M., Rampone E., Ciccarelli A., Bianco A.R., Abbruzzese A., Lupoli G. (2000). Slow release lanreotide in combination with interferon-alpha2b in the treatment of symptomatic advanced medullary thyroid carcinoma. J. Clin. Endocrinol. Metab..

[28] Cano J.M., Galan R., Lopez R. (2017). Recurrent Metastatic Medullary Thyroid Carcinoma: A Case of Sustained Response to Prolonged Treatment with Somatostatin Analogues. Thyroid. Off. J. Am. Thyroid. Assoc..

[29] Hanna G.J., Busaidy N.L., Chau N.G., Wirth L.J., Barletta J.A., Calles A., Haddad R.I., Kraft S., Cabanillas M.E., Rabinowits G. (2018). Genomic correlates of response to everolimus in aggressive radioiodine-refractory thyroid cancer: A phase II study. Clin. Cancer Res. Off. J. Am. Assoc. Cancer Res..

[30] Schneider T.C., de Wit D., Links T.P., van Erp N.P., van der Hoeven J.J., Gelderblom H., van Wezel T., van Eijk R., Morreau H., Guchelaar H.J. (2015). Beneficial Effects of the mTOR Inhibitor Everolimus in Patients with Advanced Medullary Thyroid Carcinoma: Subgroup Results of a Phase II Trial. Int. J. Endocrinol..

[31] Schneider T.C., de Wit D., Links T.P., van Erp N.P., van der Hoeven J.J., Gelderblom H., Roozen I.C., Bos M., Corver W.E., van Wezel T. (2017). Everolimus in Patients With Advanced Follicular-Derived Thyroid Cancer: Results of a Phase II Clinical Trial. J. Clin. Endocrinol. Metab..

