# Supplementary material for: A Multicenter Randomized Phase II Study of Single Agent Efficacy and Optimal Combination Sequence of Everolimus and Pasireotide LAR in Advanced Thyroid Cancer
Source: Cancers (Basel). 2022 May 26;14(11):2639. doi: 10.3390/cancers14112639 (PMC9179856; doi:10.3390/cancers14112639)
Supplement: Supplementary file 1 [file cancers-14-02639-s001.zip › cancers-1717549-supplementary.pdf]

cancers

MDPI

Supplementary Materials of “A Multicenter Randomized Phase II Study of Single Agent Efficacy and Optimal Combination Sequence of Everolimus and Pasireotide LAR in Advanced Thyroid Cancer”

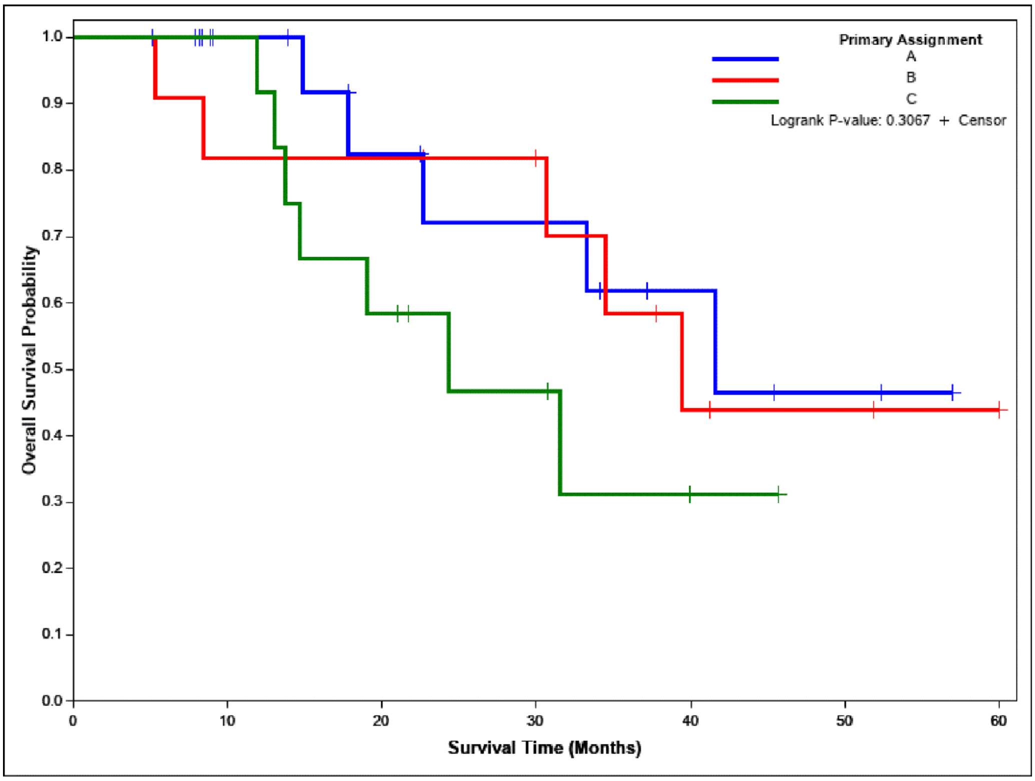

Figure S1. Overall survival was not significantly different between the three arms of the study although it was numerically higher for patients enrolled into arm A or B in comparison to arm C. Median OS of 41.6 (17.8, NA), 39.4 (8.4, NA), and 24.3 (13.1, NA) months, respectively. 1- Year OS rate was 100.0% (NA, NA), 81.8% (44.7%, 95.1%), and 91.7% (53.9%, 98.8%) respectively for arms A, B, and C while 2-year OS rate was 72.2% (35.7%, 90.2%), 81.8% (44.7%, 95.1%), and 58.3% (27.0%, 80.1%) for the three arms.

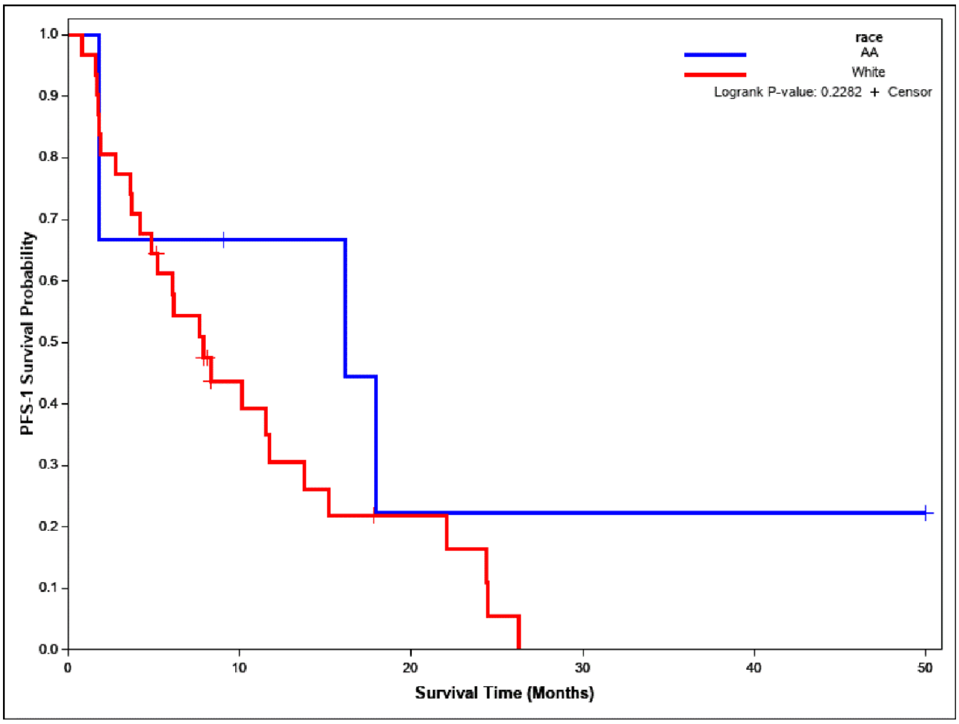

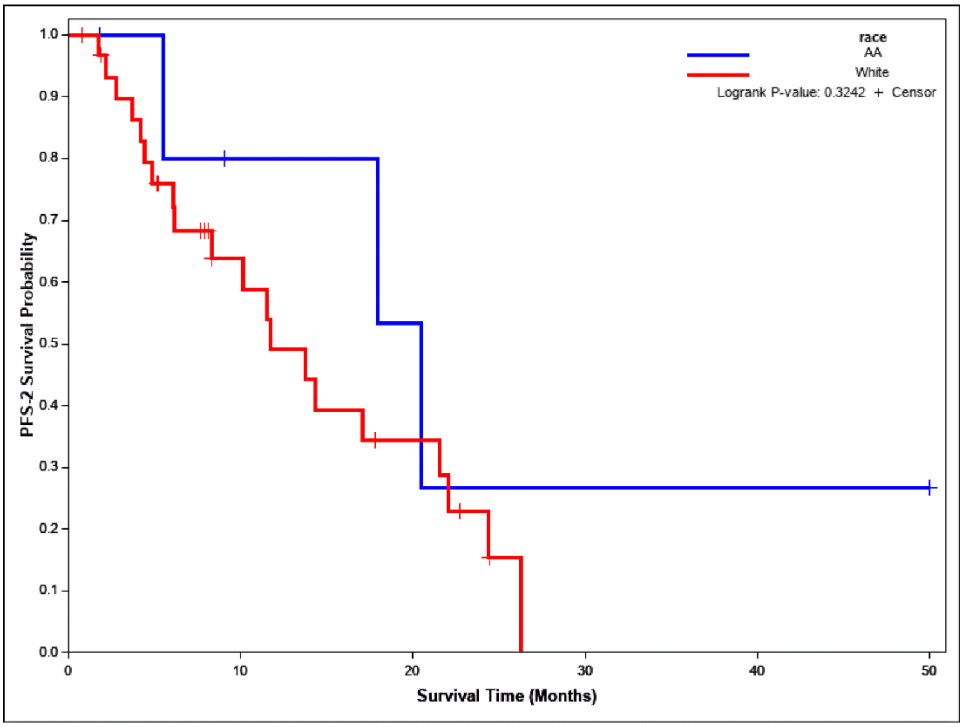

Figure S2. Kaplan-Meier curves for PFS1 and PFS2 showing no significant difference in efficacy by race. Median and 1-year PFS1 rates of 16.1 (1.8, NA) versus 7.9 (4.2, 11.7) and 66.7% (19.5%, 90.4%) vs. 30.5% (14.5%, 48.2%) for African-Americans and Whites, respectively. Similarly, median and 1-year PFS2 rates were comparable at 20.5 (5.5, NA) vs. 11.7 (6.2, 21.6) and 80.0% (20.4%, 96.9%) vs. 49.1% (28.1%, 67.1%) for African-Americans and Whites, respectively.

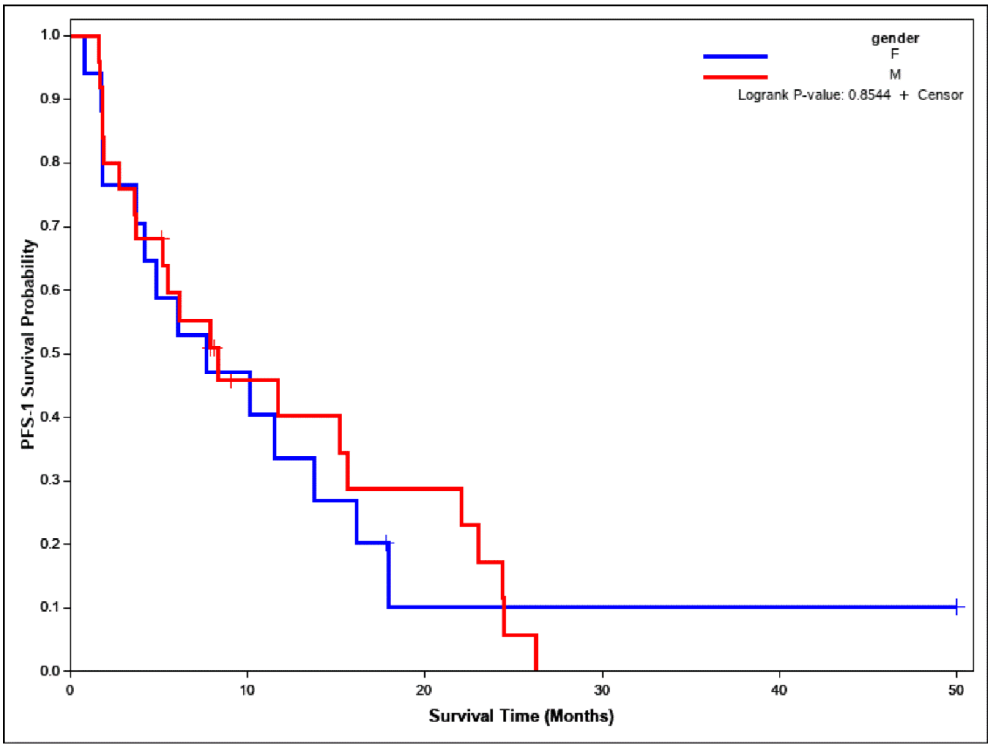

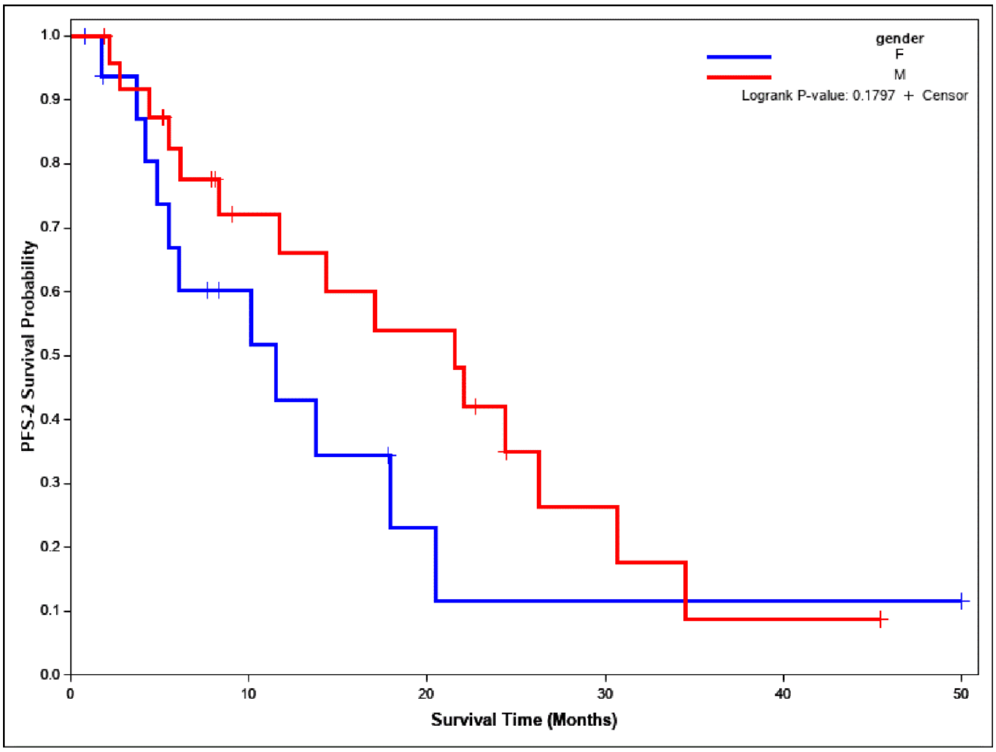

Figure S3. Kaplan-Meier curves for PFS1 and PFS2 showing no statistically significant difference in efficacy by gender. Median and 1-year PFS1 rates of 7.7 (1.8, 13.8) months versus 8.3 (3.7, 15.6) months and 33.6% (12.9%, 56.0%) vs. 40.2% (20.2%, 59.5%) for females and males, respectively. The median and 1-year PFS2 rates were also not significantly different although there was a strong trend in favor of male patients; 11.5 (4.8, 18) versus 21.6 (8.3, 26.3) months; and 43.0% (17.1%, 66.9%) versus 66.1% (41.0%, 82.4%) for females and males, respectively.

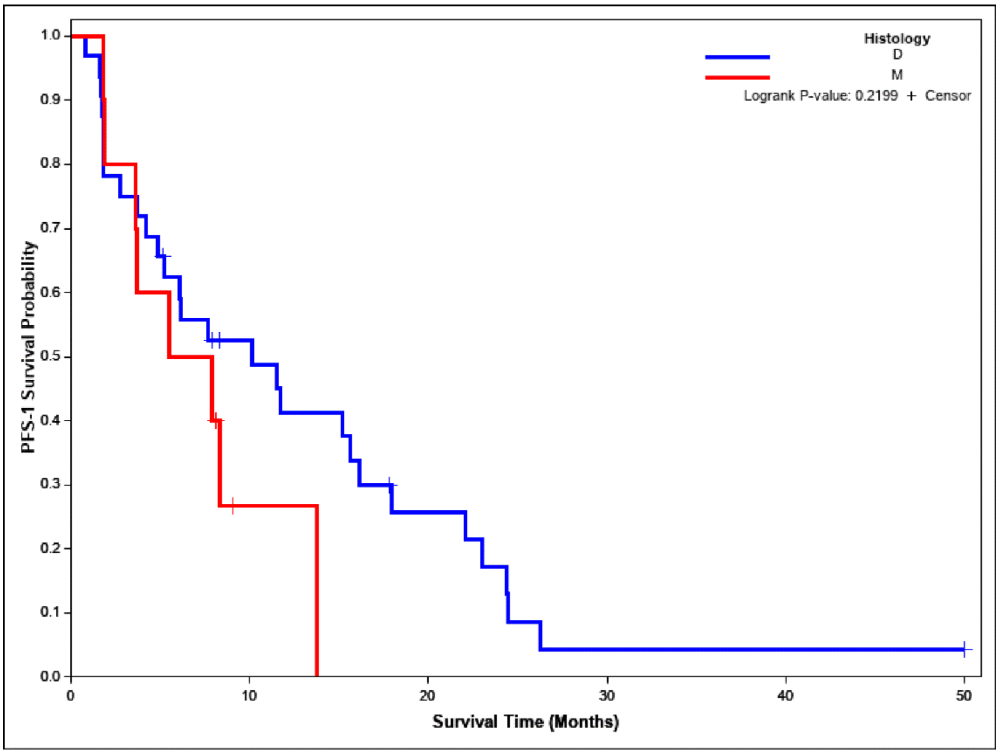

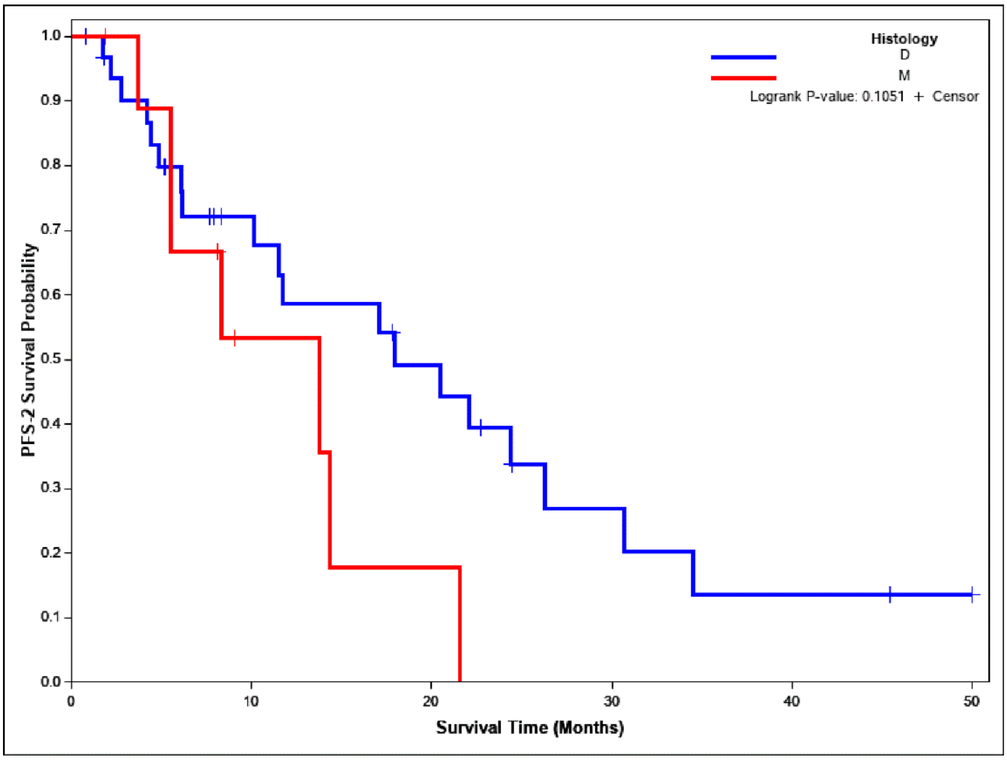

Figure S4. There was no statistically significant difference in efficacy by tumor histology although PFS1 and PFS2 were numerically higher with differentiated thyroid cancer. The median and 1-year PFS1 rates were 10.1 (4.2, 16.1) versus 6.7 (1.8, 13.8) months; and 41.3% (23.7%, 58.0%) versus 26.7% (4.8%, 56.3%), respectively, for DTC (D) and MTC (M) subtypes. A similar trend was noted for PFS2 where the median and 1-year rates were 18 (10.1, 26.3) versus 13.8 (3.7, 21.6); and 58.6% (37.2%, 74.9%) versus 53.3% (17.7%, 79.6%) for DTC (D) and MTC (M), respectively.
